# The effect of pollen monodiets on fat body morphology parameters and energy substrate levels in the fat body and hemolymph of *Apis mellifera* L. workers

**DOI:** 10.1038/s41598-024-64598-0

**Published:** 2024-07-02

**Authors:** Maciej Sylwester Bryś, Bernard Staniec, Aneta Strachecka

**Affiliations:** 1 Department of Invertebrate Ecophysiology and Experimental Biology, University of Life Sciences in Lublin, Lublin, Poland; 2 Departament of Zoology and Nature Protection, Maria Curie-Sklodowska University, Lublin, Poland

**Keywords:** Nutrition, Diets, Biomarkers, Biochemical pathway, Cell metabolism, Honeybee, Zoology, Entomology, Metabolism

## Abstract

Human activities associated with large-scale farms and the monocultures expose honey bees to one type of food. Moreover, there is an ongoing decline of plant species producing pollen and nectar in Europe. A poorly balanced diet affects a number of processes occurring in a bee’s body. The fat body and hemolymph are the tissues that participate in all of them. Therefore, the aim of our study was to determine the effect of hazel, pine, rapeseed, buckwheat, phacelia and goldenrod pollen on the morphological parameters of fat body trophocytes, the diameters of cell nuclei in oenocytes and the concentrations of compounds involved in energy metabolism (glucose, glycogen, triglycerides and protein). In the cage tests, the bees were fed from the first day of life with sugar candy (control group) or candy with a 10% addition of one of the 6 pollen types. Hemolymph and fat body from various locations were collected from 1-, 7- and 14-day-old workers. Pollen produced by plant species such as hazel and pine increased glucose concentrations in the bee tissues, especially in the hemolymph. It can therefore be concluded that they are valuable sources of energy (in the form of simple carbohydrates) which are quickly used by bees. Pollen from plants blooming in the summer and autumn increased the concentrations of proteins, glycogen and triglycerides in the fat body, especially that from the third tergite. The accumulation of these compounds was associated with an increased the length and width of trophocytes as well as with enhanced metabolic activity, which was evidenced in the increasing diameter of oenocyte cell nuclei. It seems a balanced multi-pollen diet is more valuable for bees, but it is important to understand the effects of the particular pollen types in the context of a mono-diet. In the future, this will make it possible to produce mixtures that can ensure homeostasis in the apian body.

## Introduction

The causes of the decline in honey bee populations are to be sought in the disruption of immune mechanisms, which are weakened by pathogens, parasites and, above all, human errors, e.g. treating bee colonies with deleterious substances, adulterating beeswax with stearin and paraffin and environmental chemisation^1–4^. In recent years, a serious problem for pollinating insects is the nutritional stress resulting from the lack of sufficient food and/or the lack of a balanced diet. This is the upshot of the expansion of intensive farming, crop acreage, monocultures and decreasing plant diversity (depending on the season and landscape composition)^5^. The nutritional quantity and quality of pollen and nectar can differ between plant species^6^. A poorly balanced diet and periods of starvation contribute to losses in bee colonies in Europe and around the world^7,8^. Already in the second half of the twentieth century, supplements based on soy protein, yolk powder, and fish meal were being added to bee diets, but they failed to fully replace flower pollen^9,10^. Mixed pollen is considered the most valuable for honey bees^11^ due to the great diversity and composition of the nutrients it contains. Alaux et al.^12^ identified an increased expression of several genes, relevant to oxidative stress prevention of, longevity and immune functions in bees that consumed pollen compared to those fed sugar syrup.

Pollen consumption depends not only on the age of bees and the tasks they undertake, but also on the size of the colony and the available plant sources of pollen and nectar within the flight range^13^. Pollen loads are the main source of not only amino acids, saturated and unsaturated fatty acids, vitamins, minerals but also carbohydrates^11^. Nectar provides mainly simple carbohydrates^5^. Assuming a pollen load a strong bee colony needs about 50 kg of pollen per year^14^. In addition, 6–9-day-old nurses also require large amounts of pollen in order to activate the larva feeding mechanisms and also need a source of protein to produce the jelly to feed the larvae^15,16^. As workers age, they mainly switch to food rich in simple carbohydrates, i.e. nectar, because they are no longer able to digest pollen grains. Pollen deficiency in bees results in the consumption of the glycogen stores accumulated in the trophocytes of the fat body. In such a case, it is necessary for the beekeeper to provide, for example, sugar candies with pollen, which will important nutrients to rebuild the reserves in the fat body^17^. Pollen loads are also a source of lipids stored in trophocytes in the form of triglycerides.

In spring, the first food for bees in Central and Eastern Europe is hazel pollen. Even though it is a wind-pollinated and non-nectariferous plant, bees make use of its potential^18^. Another such pollen is that of pine trees, well-known as a “natural micro-nutrient bank”, which contains the male spores of *Pinus massoniana* Lamb., *Pinus tabuliformis* Carr. and other plants of the same genus. Rich in nutrients and bioactive compounds, it can provide the necessary support for the bee body’s normal functioning and metabolic regulation. Moreover, it has strong anti-ageing, antioxidant and immunomodulatory properties^19^. In May and June, rapeseed blooms intensively. It is widely cultivated and in many cases constitutes the basic and quantitatively dominant food resource for honey bees^20^. Rapeseed produces significant amounts of both pollen and nectar, which makes it a very attractive food source for honey bees^21^. The protein content in rapeseed pollen is approximately 27%^22^. Phacelia, which blooms in late June–early July, is a plant sown by beekeepers to supplement the bees’ nutritional base. The great potential phacelia, well-known for its antioxidant, antibacterial and anti-inflammatory properties, lies in the combined provision of nectar and pollen^23,24^. Giovanetti et al.^25^ found a 25% phacelia pollen protein content in fresh beebread. This highly nutritional pollen, reduces the bees’ sensitivity to pesticides. In July, bees also benefit from buckwheat, which blooms for 3–4 weeks. Being a pseudocereal, buckwheat is ecologically undemanding and boasts a wealth of nutrients^26^. Buckwheat pollen contains phenolic acids, including galloylated derivatives and procyanidin dimers, which are antioxidants^27^. At the beginning of August, goldenrod begins to bloom and continues to do so for about 6 weeks. Individual flowers of *Solidago* sp. do not produce large amounts of nectar and pollen. However, the plants grow in large clusters, forming a mass flower display. The average pollen yield is 30.7 kg/ha^28^.

An interesting phenomenon is the negative feedback effect associated with pollen collection. Honey bee workers make use of the plants available around the apiary. If one particular pollen accumulates to a high level, it inhibits foragers from collecting other pollen types^29^. This is of high practical significance, because if the bees quickly fill the honey combs with just one type of pollen loads (e.g. rapseed pollen), the appearance of new, more attractive pollen produced by different plants will soon not encourage the bees to fly enmasse.

Enzymes produced in the head glands, the so-called glycoside hydrolases, help convert nectar into honey^30^. The actual process of digestion of pollen grains begins only in the midgut, because proteins, lipids and some carbohydrates are broken down in this section of the digestive tract. Pollen consumption stimulates the secretion of proteases, which are analogues of mammalian trypsin and chymotrypsin^31,32^. Proteolytic enzymes break down polypeptide chains. Deamination leads to the formation of alpha-keto acids which are transformed into glucose, the simplest sugar, or intermediate products for the formation of other compounds. A properly functioning midgut ensures the flow of nutrients to the hemolymph. This tissue distributes nutrients among the organs. In addition, it is an energy reserve for quick use of ingredients, for example during the flight, and participates in the defense against pathogens^33^. The circulatory system being open, the circulating hemolymph washes the fat body. The honeybee’s fat body is a very important tissue with regard to metabolic carbohydrates, proteins and lipids. In this tissue the process of glycogenesis occurs, i.e. the transformation of glucose into glycogen. This form is stored in the fat body as an energy reserve. On the other hand there are synthesis, transport, accumulation and release of proteins, lipids and carbohydrates take place.

The fat body consists of two main types of cells—trophocytes and oenocytes. Trophocytes in addition to the storage function, secrete and detoxify organic substances. Oenocytes have vacuoles containing drops and granules of lipids, proteins, and glycogen. In turn, the sizes of the nuclei of the oenocytes are indicative of the nuclear metabolic activities^30,34^. The quantity and quality of nutrients stored in pollen grains will, to a greater or lesser extent, be incorporated into the cells of the fat body. Nutrients stored in the fat body in the form of glycogen and triglycerides are necessary to maintain homeostasis. Thanks to this, trophocytes and oenocytes will produce proteins, including immune proteins and hormones, and synthesize biological compounds.

Therefore, we hypothesized that spring, summer and autumn pollen differently affects the morphology and metabolism of the fat body and hemolymph. Pollen from plants such as rapeseed, buckwheat, phacelia and goldenrod has a greater impact on the metabolism of energy reserves in the form of glucose, triglycerides and glycogen, and also protein synthesis than anemophilous pollen due to its different nutritional composition. The aim of the research was to determine the influence of a mono-diet of pollen from specific plants (hazel, pine, rapeseed, buckwheat, phacelia, goldenrod) on the morphological parameters of the fat body and energy metabolism (glucose, glycogen, triglycerides and protein) in this tissue and in the hemolymph in *Apis mellifera* L.

## Materials and methods

### Pollen load collection, pollen analysis and preparation of sugar candy with different pollen loads

From May to September 2022 mixed bee pollen loads were collected using pollen traps located at hives of local beekeepers in the Lublin region. The areas from where the pollen was obtained are considered unpolluted and free from heavy metals and pesticides. Until classification, the pollen was stored at a temperature of 20 °C. Pollen loads were then manually color-sorted to obtain the dominant pollen in each sample. Granules of the same color were pooled. For pollen analysis, 3.5 g of bee pollen were collected and suspended in a 1:1 10 ml solution of distilled water with glycerin. The microscopic preparations were prepared from such suspensions/solutions according to the method of Filipiak et al. (2022)^35^. The results of the botanical analysis of pollen samples of uniform colors are presented in Table 1.

**Table 1:** Frequency (%) of the most widely represented pollen species in the bee diets.

No	Pollen loads samples	Dominant pollen species	Frequency [%]	Other species
1	Hazel	*Corylus* sp.	98	*Prunus* sp.
2	Pine	*Pinus sylvestris* L.	98	*Taraxacum* sp. *Brassica napus*
3	Rapeseed	*Brassica napus* L.	92	*Juglans* sp. *Quercus* sp. *Carex* sp. *Pinus sylvestris* *Taraxacum* sp. *Prunus* sp.
4	Buckwheat	*Fagopyrum esculentum* Moench	75	*Trifolium* sp. *Centaurea* sp. *Vicia* sp. Brassicaceae
5	Phacelia	*Phacelia tanacetifolia* Benth	99	*Centaurea* sp.
6	Goldenrod	*Solidago* sp.	95	*Anthriscus* sp. *Fagopyrum esculentum* *Centaurea* sp.

The sugar candy prepared from 500 ml water and 2.3 kg of white granulated sugar, was divided into 7 parts. No pollen was added to the first part and this candy was fed to bees in the control group. Specific, previously micronized, pollen particles (according to Table 1) were then added to every other part at a concentration of 10% and the bees were fed with them *ad libidum* (6 groups). According to Altaye et al.^36^, worker bees need 10 mg of protein and 90 mg of carbohydrates per 100 mg diets.

### Obtaining 1-day-old bees and experimental cages

One-day-old bees were obtained from 10 colonies in an apiary that belongs to University of Life Sciences in Lublin (51° 22′ N, 22° 63′ E), Poland. The bees in the colonies were in good health. The queens were confined for 12 h in a queen-excluder comb-cage containing one empty comb to lay eggs. Twenty days later, after the queens had laid their eggs, the combs were transferred to an incubator, where the 1-day-old workers emerged. These workers were randomly placed in 105 wooden cages (100 bees per cage) and divided into seven groups (15 cages each). Additionally, 45 freshly emerged workers were collected for laboratory analyses.

All the cages with bees were kept in controlled conditions at 35 °C and 65% relative humidity. The control group was fed sugar candy only while the other groups were fed candy containing the addition of 10% of a specific pollen, such as hazel, pine, rapeseed, buckwheat, phacelia or goldenrod. Thus, 7 groups were formed, the feeding of which began on the first day of the workers’ lives. Throughout the entire experimental period, diets and water were replaced every two days and dead individuals were removed from the cages. In each group, 7- and 14-day old workers were collected from the cages for laboratory analysis (2–4 bees per cage). This yielded the following database: 40–55 workers × 7 groups × 2 sampling + 45 1-day old workers.

### Laboratory analyses

#### Hemolymph collection

Fresh hemolymph was individually taken from between the third and fourth tergite of living workers to a glass capillary (20 µl; ‘end to end’ type; without anticoagulant; Medlab Products, Raszyn, Poland) according to the Łoś and Strachecka’s^37^ method. Prior to hemolymph collection, each bee was punctured with a sterile needle in the abdomen. Hemolymph from one bee was collected into one sterile Eppendorf tube containing 200 µl of ice-cooled 0.6% NaCl and then immediately refrigerated at − 25 °C for further biochemical analyses.

### Fat body analysis

Immediately after the hemolymph was collected, the fat bodies from the sternites and from the third, fourth, fifth, sixth, and seventh tergites were dissected under a Stereo Zoom Microscope in 0.6% *natrium chloratum*. Moreover, the fat bodies from these tergites and sternites were divided in half the first half was used to make a microscopic preparation and the second half (from the third and fifth tergites and sternites) was used in the biochemical analyses. The choice of these three locations for biochemical analyses resulted from Strachecka’s et al.^34^ previous research, which proved that the fat body from these three locations is the most metabolically active.

The fat bodies from the first half were placed on glass slides in 0.6% *natrium chloratum* (pro inj.) and covered with cover-glasses. The microscopic preparations were observed with Camera Olympus DP72 (Microscope Olympus BX61; magnification 40×) with a DIC attachment and the fat body cells were measured according to Strachecka et al.’s^34^ method. This method enables (undistorted) visualization of living preparation according to Strachecka et al.^38^. The following measurements made under the microscope are the maximum in 100 randomly selected cells:

- the length and width of the trophocytes for each segment,
- diameter of the oenocyte nuclei for all segments except tergite 3, because honey bee workers do not have oenocytes in this place or they are sporadic. We also confirmed these observations in our experiment. Measurements were made according to Strachecka et al.’s^34^ method. Eight people were involved in the preparation and measurement of the length and width of trophocytes and oenocytes of the fat body.

The fat bodies from the second half were collected in sterile Eppendorf tubes, containing 25 μl of ice-cooled 0.6% NaCl. Next, the tissues were homogenised at 4 °C and centrifuged for 1 min at 3000 g. The supernatants were immediately refrigerated at − 40 °C for further biochemical analyses.

### Biochemical analysis

The following parameters were determined in the supernatants from the fat bodies and hemolymph solutions:

- glucose and triglyceride concentrations were determined using the Alpha Diagnostics reagent kit (Glucose DST G6620-100; G6620-500; Triglycerides DST T6630-100; T6630-500) according to the manufacturer’s instructions;
- total protein concentrations were determined using the Lowry method modified by Schacterle and Pollack^39^;
- glycogen concentrations were determined colorimetrically using the Glycogen Assay Kit (Sigma Aldrich, USA, No. MAK016).

### Statistical analyses

The data were analyzed using Statistica formulas (TIBCO Software, Palo Alto, CA, USA), 13.3 (2017) version for Windows—StatSoft Inc., Tulsa, OK, USA. The normal distribution of data was analyzed using the Shapiro–Wilk test (*p* > 0.05). In order to compare the measurements under the microscope and the biochemical parameters among the workers in the seven groups, a mixed model two-way and three-way ANOVAs were used. If a difference among the groups was statistically significant, the ANOVA procedure was followed with multiple comparison testing using the post hoc Tukey HSD test. In the case of correlations determined with a distribution other than normal (oenocyte diameter), the Kruskal–Wallis test was used.

## Results

The fat body in all the groups of bees fed with sugar candy with the addition of 10% bee pollen was better developed than in those fed with sugar candy only. Regardless of the pollen intake, the bees’ fat body cells had greater energy reserves in the form glucose, glycogen, triglycerides and protein than those of the bees in the control group. The values of these biochemical parameters were usually higher in bees fed with candy + pollen. Increased metabolic activity is associated with larger trophocyte sizes and oenocyte nucleus diameters in each fat body segment.

### Length of trophocytes

Bees fed sugar candy with the pollen addition tended to have longer trophocytes than those fed candy only; exceptions were some trophocytes in bees fed with candy and hazel (in 7-day-old workers—in the third and fourth tergites) and pine pollen (in 7- and 14-day-old workers—in the third to seventh tergites) (Table 2). When comparing 7-day-old bees from different groups, those fed with rapeseed pollen candy had the longest trophocytes. Overall, the 14-day-old bees fed with candy to which rapeseed, phacelia and goldenrod pollen was added had the longest trophocytes.

**Table 2:** Length of trophocytes (μm) in the fat body from different locations in the 1-, 7- and 14-day-old workers (n = 40–55 bees per group) fed sugar candy only (control group) and in those fed sugar candy with various pollen additions.

					Pollen added to sugar candy											
Day of life	Localization tissues		Control		Hazel		Pine		Rape		Buckwheat		P hacelia		Goldenrod	
			Mean	SD	Mean	SD	Mean	SD	Mean	SD	Mean	SD	Mean	SD	Mean	SD
1	Fat body	Sternite	30.69	3.03												
		Tergite 3	50.20	5.47												
		Tergite 4	45.86	7.27												
		Tergite 5	46.02	8.87												
		Tergite 6	41.32	13.62												
		Tergite 7	48.82	10.36												
7	Fat body	Sternite	39.26	2.55	52.17	3.57	40.12	10.84	52.02	8.21	92.36	5.54	48.70	10.29	44.09	8.71
		Tergite 3	57.82	5.47	44.31	3.57	43.19	7.12	82.46	7.58	55.10	4.47	66.46	5.62	41.77	10.05
		Tergite 4	56.92	7.27	48.68	8.24	50.00	5.13	97.05	14.19	72.25	9.65	65.35	8.02	50.35	15.09
		Tergite 5	52.16	8.87	59.72	3.95	40.58	4.78	106.27	18.13	73.21	4.48	56.71	6.15	54.19	8.82
		Tergite 6	50.68	13.62	48.27	4.65	37.87	4.95	117.21	15.07	47.99	9.63	67.82	9.31	56.94	12.29
		Tergite 7	56.65	10.36	69.01	8.24	44.90	3.86	49.41	11.30	69.86	7.84	65.31	10.99	85.19	12.77
14	Fat body	Sternite	39.43	2.74	80.98	6.63	50.53	6.54	83.85	13.11	90.26	6.61	162.00	4.69	97.29	14.11
		Tergite 3	57.53	5.40	69.86	5.42	37.79	7.71	109.30	14.54	75.25	7.71	137.37	5.11	142.01	11.43
		Tergite 4	55.15	7.34	125.29	5.51	53.70	3.21	152.63	19.52	79.59	9.60	110.82	10.05	130.12	8.64
		Tergite 5	54.38	8.76	84.24	4.47	47.10	3.74	80.41	6.34	71.59	10.12	115.37	15.06	113.12	15.69
		Tergite 6	50.23	13.18	83.90	3.32	39.18	7.41	101.30	10.99	75.72	11.09	154.20	8.82	133.64	11.51
		Tergite 7	52.69	10.18	82.65	9.52	47.63	8.84	86.73	14.87	54.93	8.26	134.81	12.29	131.13	9.47
Three-Way Anova	Group F_(6,706)_ = 576.51; *p* = 0.05															
	Localization F_(3,706)_ = 248.09; *p* = 0.049															
	Age F_(1,674)_ = 16,724.0; *p* = 0.00															
	Group * age F_(6,674)_ = 779.68; *p* = 0.05															
	Group * localization tissue F_(18,706)_ = 42.527; *p* = 0.00															
	Age * localization tissue F_(3,674)_ = 1235.5; *p* = 0.00															
	Group * localization tissue * age F_(18,674)_ = 234.90; *p* = 0.01															

### Width of trophocytes

The trophocyte widths increased with age in all the groups (Table 3). The trends in these widths were similar to those in the trophocyte lengths. The trophocyte widths in each segment in the bees fed pine pollen were similar to those in the cells of the bees in the control sample. In other groups, the bees fed with sugar candy plus added pollen tended to have wider trophocytes than those fed candy only.

**Table 3:** Width of the trophocytes (μm) in the fat body from different locations in the 1-, 7- and 14-day-old workers (n = 40–55 bees per group) fed sugar candy only (control group) and in those fed sugar candy with added pollen.

					Pollen added to sugar candy											
Day of life	Localization tissues		Control		Hazel		Pine		Rape		Buckwheat		Phacelia		Goldenrod	
			Mean	SD	Mean	SD	Mean	SD	Mean	SD	Mean	SD	Mean	SD	Mean	SD
1	Fat body	Sternite	28.95	4.71												
		Tergite 3	37.90	5.66												
		Tergite 4	39.26	5.80												
		Tergite 5	38.23	11.58												
		Tergite 6	33.25	6.68												
		Tergite 7	37.07	4.37												
7	Fat body	Sternite	33.02	4.21	65.09	8.51	38.90	8.14	44.07	8.59	29.00	11.58	33.79	8.63	40.99	4.40
		Tergite 3	40.15	4.71	40.14	4.57	34.87	11.02	52.71	7.99	40.90	13.36	50.04	5.41	32.93	7.26
		Tergite 4	42.49	5.66	42.96	5.56	37.03	10.54	55.06	18.13	39.02	11.28	51.43	8.21	40.28	11.03
		Tergite 5	40.94	5.80	59.36	9.65	42.44	9.58	81.91	9.44	35.56	14.57	43.34	5.33	48.00	8.17
		Tergite 6	35.95	11.58	39.18	4.25	37.52	7.51	83.97	9.77	42.60	10.09	47.79	8.50	47.11	8.09
		Tergite 7	39.68	6.63	47.22	6.54	38.51	4.85	44.96	8.89	26.10	5.54	57.54	12.99	83.94	11.27
14	Fat body	Sternite	34.56	4.24	49.90	4.7	43.94	8.63	84.23	8.41	56.30	9.91	100.00	8.63	78.62	3.50
		Tergite 3	42.00	4.73	54.01	7.81	34.81	7.69	65.92	6.32	70.00	10.74	86.53	12.82	98.73	10.14
		Tergite 4	46.16	5.56	76.48	6.03	65.66	6.31	96.01	4.58	71.54	12.54	100.02	12.25	112.19	12.25
		Tergite 5	42.69	5.74	43.10	7.81	51.60	4.85	48.74	11.58	69.00	14.85	88.22	11.14	106.58	13.13
		Tergite 6	36.84	11.48	58.95	4.15	64.59	8.69	86.24	8.98	50.14	8.51	113.00	12.24	139.47	13.60
		Tergite 7	41.67	6.57	46.79	7.65	47.85	12.27	65.00	14.93	54.25	11.02	114.08	11.84	116.00	14.41
Three-Way Anova	Group F_(6,706)_ = 576.51; *p* = 0.00															
	Localization F_(3,706)_ = 248.09; *p* = 0.05															
	Age F_(1,674)_ = 16,724.0; *p* = 0.00															
	Group * age F_(6,674)_ = 779.68; *p* = 0.00															
	Group * localization tissue F_(18,706)_ = 42.527; *p* = 0.00															
	Age * localization tissue F_(3,674)_ = 1235.5; *p* = 0.00															
	Group * localization tissue * age F_(18,674)_ = 234.90; *p* = 0.01															

### Diameters of oenocyte nuclei

The diameters of oenocyte nuclei in the groups of bees fed with pollen were comparable, but statistically significantly larger than those of the nuclei in the group of bees fed with sugar candy only (Table 4). On day 7, the smallest diameter was recorded in tergites 4 and 6 of the bees fed with 10% goldenrod pollen, while the highest values were recorded in tergites 5 and 7 for rapeseed pollen. On day 14, the oenocytes of bees fed the phacelia pollen had the largest diameters in all segments compared to the other groups of bees.

**Table 4:** Diameters of the oenocyte nuclei (μm) in the fat body from different locations in the 1-, 7- and 14-day-old workers (n = 40–55 bees per group) fed sugar candy only (control group) and in those fed sugar candy with added pollen.

					Pollen added to sugar candy											
Day of life	Localization tissues		Control		Hazel		Pine		Rape		Buckwheat		Phacelia		Goldenrod	
			Mean	SD	Mean	SD	Mean	SD	Mean	SD	Mean	SD	Mean	SD	Mean	SD
1	Fat body	Sternite	10.80	0.31												
		Tergite 4	6.32	0.34												
		Tergite 5	8.58	0.24												
		Tergite 6	7.41	0.54												
		Tergite 7	8.40	0.66												
7	Fat body	Sternite	11.59	0,61	16.52	0.32	15.70	0.34	15.91	0.22	14.14	0.68	12.09	0.29	13.92	0.37
		Tergite 4	9.85	0.95	16.12	0.41	11.21	0.41	14.46	0.37	14.79	0.54	9.85	0.33	9.82	0.39
		Tergite 5	10.90	0.84	14.08	0.54	14.37	0.29	17.53	0.31	16.15	0.41	10.90	0.31	10.37	0.54
		Tergite 6	9.05	0.41	15.58	0.41	14.78	0.38	15.45	0.42	16.16	0.29	10.05	0.39	9.69	0.57
		Tergite 7	9.99	0.85	17.32	0.29	16.07	0.41	17.34	0.33	17.28	0.61	10.99	0.41	12.66	0.54
14	Fat body	Sternite	12.49	0.81	15.13	0.47	14.67	0.65	15.09	0.33	14.62	0.68	19.63	0.48	14.04	0.87
		Tergite 4	10.58	0.65	13.78	0.40	13.76	0.41	15.63	0.28	16.61	0.54	18.00	0.51	15.54	0.54
		Tergite 5	11.46	0,32	15.77	0.33	13.56	0.33	16.95	0.33	15.66	0.41	16.00	0.41	15.85	0.41
		Tergite 6	9.56	0,22	15.05	0.32	14.36	0.70	15.27	0.31	16.06	0.29	18.25	0.49	9.33	0.46
		Tergite 7	11.02	0.34	17.20	0.30	14.41	0.65	17.11	0.32	14.63	0.33	17.99	0.37	17.96	0.39
Three-Way Anova	Group F_(6,5537)_ = 386.36; *p* = 0.00															
	Localization F_(4,5537)_ = 19.677; *p* = 0.00															
	Age F_(2,557)_ = 495.63; *p* = 0.01															
	Group * age F_(5,5498)_ = 1362.7; *p* = 0.05															
	Group * localization tissue F_(24,5537)_ = 40.417; *p* = 0.05															
	Age * localization tissue F_(8,5557)_ = 15.200; *p* = 0.05															
	Group * localization tissue * age F_(23,5498)_ = 124.46; *p* = 0.05															

### Glucose concentrations

Glucose concentrations were always higher in the workers which consumed pollen (candy + 10% pollen) compared to those fed sugar candy only (Table 5). The smallest increase in glucose concentrations was caused by pollen from spring-pollinating flowers, i.e. hazel and pine, while the highest increases were observed in the case of buckwheat and phacelia. Glucose concentrations increased in all the tissues (hemolymph and fat body from three locations) along with the age of the workers, regardless of the food they consumed. The lowest values, within a specific group of workers, were observed in the hemolymph, and the highest usually in the fat body in the third tergite.

**Table 5:** Glucose concentrations (mmol/ml) in the hemolymph and fat body from different locations in the 1-, 7- and 14-day-old workers (n = 40–55 bees per group) fed sugar candy only (control group) and in those fed sugar candy with various pollen additions.

					Pollen added to sugar candy											
Day of life	Tissues/localization tissues		Control		Hazel		Pine		Rape		Buckwheat		Phacelia		Goldenrod	
			Mean	SD	Mean	SD	Mean	SD	Mean	SD	Mean	SD	Mean	SD	Mean	SD
1	Hemolymph		1.53	0.02												
	Fat body	Sternite	1.85	0.02												
		Tergite 3	2.45	0.03												
		Tergite 5	2.26	0.03												
7	Hemolymph		2.09	0.15	3.57	0.10	2.75	0.07	2.52	0.03	3.21	0.19	2.47	0.18	2.92	0.13
	Fat body	Sternite	2.42	0.00	2.64	0.03	2.60	0.06	3.17	0.19	8.48	0.19	9.54	0.38	7.47	0.26
		Tergite 3	2.47	0.13	5.67	0.05	2.75	0.05	2.92	0.17	3.64	0.21	2.95	0.15	3.85	0.19
		Tergite 5	2.94	0.04	3.0	0.04	2.98	0.10	2.99	0.07	5.84	0.58	6.42	0.29	3.64	0.64
14	Hemolymph		3.38	0.09	3.89	0.04	3.78	0.06	4.6	0.04	5.03	0.12	4.65	0.20	4.61	0.05
	Fat body	Sternite	3.46	0.04	2.64	0.04	3.78	0.04	4.77	0.02	14.7	0.38	10.44	0.10	8.15	0.05
		Tergite 3	4.55	0.05	5.47	0.21	5.07	0.10	8.06	0.02	10.54	0.35	12.13	0.06	9.53	0.04
		Tergite 5	3.84	0.02	3.94	0.04	3.87	0.01	6.15	0.02	13.39	0.18	11.06	0.03	8.89	0.09
Three-Way Anova	Group F_(6,706)_ = 576.51; *p* = 0.00															
	Localization F_(3,706)_ = 248.09; *p* = 0.00															
	Age F_(1,674)_ = 16,724.0; *p* = 0.00															
	Group * age F_(6,674)_ = 779.68; *p* = 0.02															
	Group * localization tissue F_(18,706)_ = 42.527; *p* = 0.00															
	Age * localization tissue F_(3,674)_ = 1235.5; *p* = 0.00															
	Group * localization tissue * age F_(18,674)_ = 234.90; *p* = 0.00															

### Triglyceride concentrations

The addition of pollen to the sugar candy diet increased the concentrations of triglycerides in all the tissues (Table 6). Both sternite and tergite 3 stored the largest amounts of triglycerides, regardless of the age and the study group. Buckwheat and rapeseed pollen produced the greatest increase in triglyceride concentrations compared to the control sample in the two locations above, especially on day 14.

**Table 6:** Triglyceride concentrations (mmol/ml) in the hemolymph and fat body from different location in the 1-, 7- and 14-day-old workers (n = 40–55 bees per group) fed sugar candy only (control group) and in those fed sugar candy with added pollen.

					Pollen added to sugar candy											
Day of life	Tissues/localization tissues		Control		Hazel		Pine		Rape		Buckwheat		Phacelia		Goldenrod	
			Mean	SE	Mean	SE	Mean	SE	Mean	SE	Mean	SE	Mean	SE	Mean	SE
1	Hemolymph		0.23	0.01												
	Fat body	Sternite	0.31	0.00												
		Tergite 3	0.32	0.01												
		Tergite 5	0.38	0.00												
7	Hemolymph		1.06	0.19	1.01	0.07	1.42	0.11	1.42	0.03	0.96	0.11	1.63	0.13	0.87	0.16
	Fat body	Sternite	1.36	0.09	1.16	0.02	1.24	0.02	1.06	0.07	1.76	0.02	1.65	0.04	1.8	0.02
		Tergite 3	0.94	0.15	1.21	0.03	1.14	0.02	1.46	0.05	1.51	0.06	1.64	0.04	1.49	0.05
		Tergite 5	1.06	0.07	1.14	0.02	1.21	0.05	1.46	0.02	1.42	0.06	1.53	0.04	1.53	0.05
14	Hemolymph		1.55	0.05	1.66	0.05	1.67	0.02	2.18	0.02	2.29	0.07	2.05	0.03	1.84	0.01
	Fat body	Sternite	2.09	0.03	2.2	0.04	2.16	0.03	2.94	0.13	4.24	0.17	2.55	0.03	2.36	0.03
		Tergite 3	2.47	0.05	3.17	0.09	2.94	0.06	4.54	0.17	5.51	0.26	4.65	0.22	4.14	0.26
		Tergite 5	1.85	0.02	2.03	0.09	1.9	0.03	2.56	0.04	3.37	0.21	2.37	0.03	2.06	0.02
Three-Way Anova	Group F_(6,706)_ = 126.89; *p* = 0.00															
	Localization F_(3,706)_ = 240.82; *p* = 0.00															
	Age F_(1,674)_ = 7986.0; *p* = 0.00															
	Group * age F_(6,674)_ = 176.43; *p* = 0.00															
	Group * localization tissue F_(18,706)_ = 12.590; *p* = 0.01															
	Age * localization tissue F_(3,674)_ = 613.03; *p* = 0.01															
	Group * localization tissue * age F_(18,674)_ = 25.613; *p* = 0.01															

### Protein concentrations

Protein concentrations increased with the age of the workers in most groups (with the exception of the 7-day-old workers fed with hazel and pine pollen) (Table 7). The addition of pollen from summer and autumn plants to the diet increased protein concentrations in all the tissues to the greatest extent compared to the control sample. Particularly high values of protein concentrations were observed in the fat body from the fifth tergite in the worker bees fed with buckwheat pollen.

**Table 7:** Protein concentrations (mg/ml) in the hemolymph and fat body from different locations in the 1, 7 and 14-day-old workers (n = 40–55 bees per group) fed sugar candy only (control group) and in those fed sugar candy with added pollen.

					Pollen added to sugar candy											
Day of life	Tissues/localization tissues		Control		Hazel		Pine		Rape		Buckwheat		Phacelia		Goldenrod	
			Mean	SE	Mean	SE	Mean	SE	Mean	SE	Mean	SE	Mean	SE	Mean	SE
1	Hemolymph		0.24	0.01												
	Fat body	Sternite	0.45	0.01												
		Tergite 3	0.45	0.01												
		Tergite 5	0.40	0.01												
7	Hemolymph		0.27	0.00	0.34	0.00	0.32	0.02	0.47	0.00	0.52	0.04	0.46	0.00	0.42	0.00
	Fat body	Sternite	0.42	0.00	0.44	0.00	0.44	0.01	0.85	0.00	0.96	0.01	0.60	0.00	0.51	0.00
		Tergite 3	0.41	0.00	0.40	0.00	0.41	0.00	0.42	0.00	0.51	0.01	0.42	0.00	0.46	0.00
		Tergite 5	0.53	0.00	0.60	0.00	0.57	0.02	0.61	0.00	0.75	0.00	0.57	0.00	0.54	0.00
14	Hemolymph		0.54	0.00	0.58	0.00	0.59	0.02	0.64	0.00	0.65	0.02	0.73	0.00	0.68	0.00
	Fat body	Sternite	1.06	0.00	1.39	0.00	1.18	0.02	1.55	0.00	1.99	0.02	1.98	0.00	1.85	0.00
		Tergite 3	0.85	0.01	0.93	0.02	0.89	0.02	1.07	0.00	1.93	0.02	1.75	0.01	1.36	0.00
		Tergite 5	0.53	0.00	0.94	0.02	0.88	0.02	0.95	0.00	3.12	0.02	1.21	0.01	0.96	0.00
Three-Way Anova	Group F_(6,706)_ = 126.89; *p* = 0.05															
	Localization F_(3,706)_ = 240.82; *p* = 0.05															
	Age F_(1,674)_ = 7986.0; *p* = 0.05															
	Group * age F_(6,674)_ = 176.43; *p* = 0.05															
	Group * localization tissue F_(18,706)_ = 12.590; *p* = 0.05															
	Age * localization tissue F_(3,674)_ = 613.03; *p* = 0.05															
	Group * localization tissue * age F_(18,674)_ = 25.613; *p* = 0.05															

### Glycogen concentrations

The glycogen concentrations in the bees fed with sugar candy only (control group) and with candy plus hazel and pine pollen were similar in all the tissues (Table 8). However, the other bee groups (fed with rapeseed, phacelia, buckwheat and goldenrod pollen) were characterized by higher glycogen concentrations. As in the case of the triglyceride concentrations, the fat body in the sternites and tergites 3 contained higher glycogen concentrations than in all the tissue locations.

**Table 8:** Glycogen concentrations (µg/µl) in the hemolymph and fat body from different locations in the 1, 7 and 14-day-old workers (n = 40–55 bees per group) fed sugar candy only (control group) and in those fed sugar candy with added pollen.

					Pollen added to sugar candy											
Day of life	Tissues/localization tissues		Control		Hazel		Pine		Rape		Buckwheat		Phacelia		Goldenrod	
			Mean	SE	Mean	SE	Mean	SE	Mean	SE	Mean	SE	Mean	SE	Mean	SE
1	Hemolymph		0.02	0.00												
	Fat body	Sternite	0.05	0.00												
		Tergite 3	0.06	0.00												
		Tergite 5	0.02	0.00												
7	Hemolymph		0.02	0.00	0.04	0.00	0.04	0.00	0.15	0.02	0.06	0.00	0.15	0.02	0.14	0.01
	Fat body	Sternite	0.08	0.00	0.11	0.00	0.09	0.00	0.15	0.02	0.24	0.00	0.16	0.02	0.17	0.01
		Tergite 3	0.09	0.00	0.09	0.00	0.1	0.00	0.23	0.02	0.32	0.00	0.24	0.02	0.25	0.02
		Tergite 5	0.03	0.00	0.05	0.00	0.04	0.00	0.08	0.02	0.08	0.00	0.10	0.02	0.09	0.02
14	Hemolymph		0.03	0.00	0.05	0.00	0.06	0.00	0.20	0.02	0.09	0.00	0.23	0.02	0.09	0.01
	Fat body	Sternite	0.10	0.00	0.22	0.00	0.16	0.00	0.16	0.02	0.25	0.00	0.19	0.02	0.22	0.01
		Tergite 3	0.15	0.00	0.14	0.00	0.16	0.00	0.27	0.02	0.34	0.00	0.32	0.02	0.33	0.02
		Tergite 5	0.04	0.00	0.07	0.00	0.05	0.00	0.09	0.02	0.14	0.00	0.10	0.02	0.15	0.02
Three-Way Anova	Group F_(6,571)_ = 190.24; *p* = 0.04															
	Localization F_(3,571_ = 402.62; *p* = 0.01															
	Age F_(1,539)_ = 247.15; *p* = 0.00															
	Group * age F_(6,539)_ = 1.093; *p* = 0.251															
	Group * localization tissue F_(18,571)_ = 24.398; *p* = 0.05															
	Age * localization tissue F_(3,539)_ = 18.854; *p* = 0.05															
	Group * localization tissue * age F_(18,539)_ = 7.8998; *p* = 0.05															

Explanations: value for SE: Control: 0.00226–0.00362; Hazel: 0.00162–0.00198, Pine: 0.00214–0.00288; Buckwheat: 0.00246–0.00222.

## Discussion

The honey bee exhibits floral fidelity, which consists in visiting mainly one plant species, which is why the pollen load composition is almost uniform^40^. Consequently, we used pollen traps to obtain pollen collected by the bee workers^15,16^. Other scientists have applied a similar methodology using pollen loads from traps in feeding experiments^5,41,42^. It is possible to obtain fresh pollen grains directly from plants, but manual collection is difficult due to the fact that pollen is available in small amounts. This method is effective only for plants with large flowers producing a lot of pollen. The method of combing corn pollen is used to study the total protein content in the hemolymph of insects^43^. However, in the case of the pollen produced by the plants in our experiment, this is mostly impossible because, unlike wind-pollinated plants, rapeseed, phacelia, goldenrod, and buckwheat produce only small amounts of pollen. In order to use the same methodology, hazel and pine pollen were also collected from traps installed in front of the hives. Forager bees mix pollen with saliva, nectar and honey. The pollen grains brought to the hive also contain sugar and enzymes of animal origin. The amount of nectar and honey added to form pollen grains varies, but can be up to 50% of dry matter^16^.

**Figure 1:**
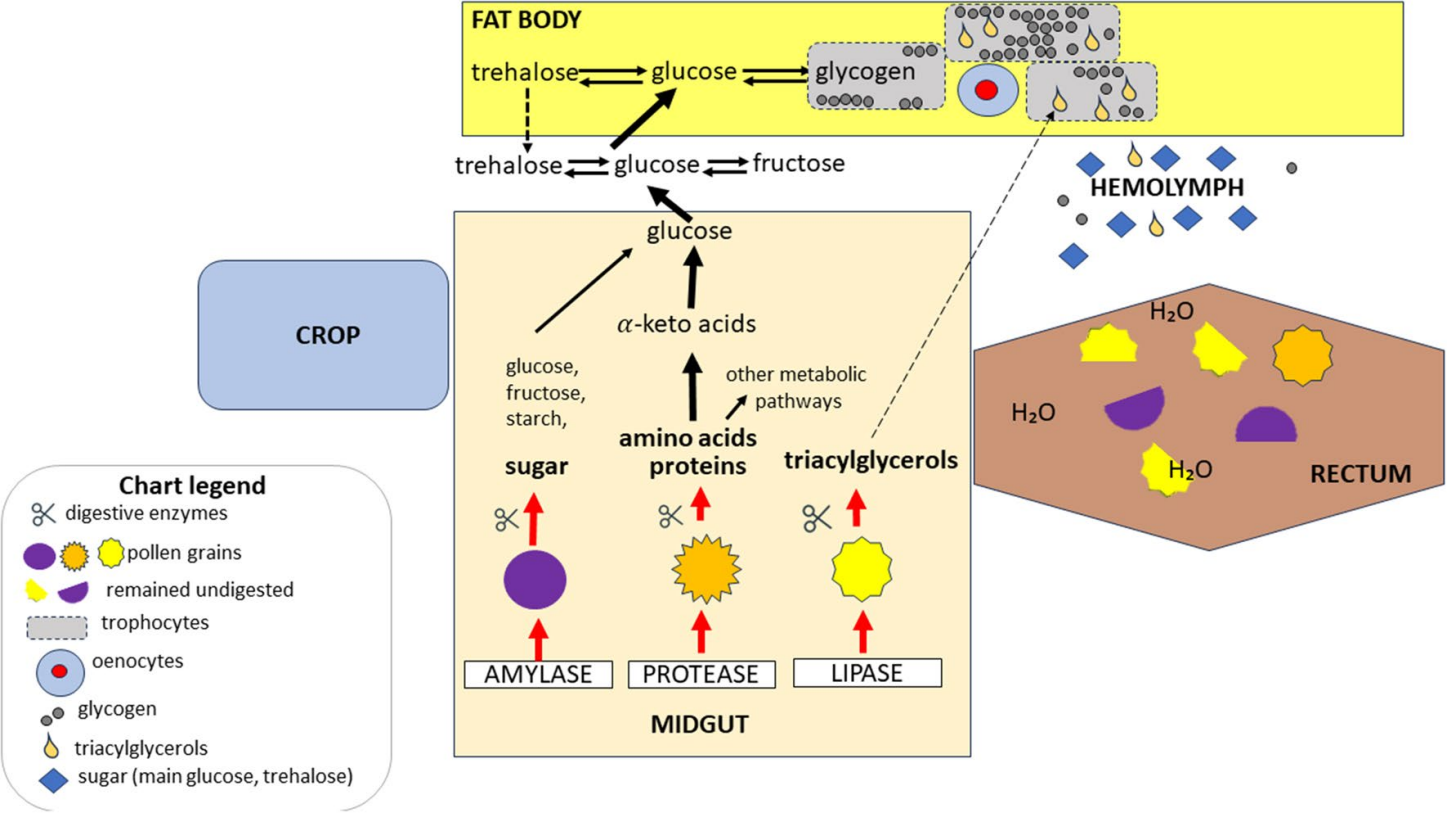
Pollen digestion: the main metabolic pathway, including the function of the fat body (orig.)

Pollen provides bee colonies mainly with protein. This parameter, together with the amino acid composition, is the basic measure of pollen quality^16^. De Grandi Hoffman et al.^44^, found that spring and autumn pollen mixtures had similar total protein concentrations. Plant pollen from these seasons differs in its amino acid composition. Spring pollen, e.g. from *Brassica*, contains relatively high concentrations of tryptophan, valine, glutamine and serine. These amino acids are components of apisimin, an alpha-helical peptide that promotes the formation of royal jelly^45^. Autumn plants produce pollen rich in proline and hydroxyproline, amino acids that support thermoregulation related to muscle vibration that enables the bees to survive the winter. In our experiment, we noticed that the bees fed with sugar candy supplemented with hazel, pine, rapeseed, phacelia, buckwheat and goldenrod pollen had higher protein concentrations in the fat body and hemolymph compared to those fed with candy only (control group). This is due to the fact that the plants have different nutrient contents^46^. Chang et al.^41^ and Breygina et al.^47^ showed that rapeseed, pear and apricot pollen play an important role in amino acid metabolic pathways and their concentrations decrease with the age of bees kept in cages. These plants are grown in China on large areas and are classified as monocultures. In turn, we showed that the protein concentrations increased with the age of the workers fed rape, buckwheat, phacelia and goldenrod pollen. These proteins in question are immune proteins, enzyme proteins, structural proteins, etc. According to Paes-de-Oliveira et al.^48^ one of such proteins is vitellogenin produced by trophocytes. Presumably, the larger these cells are, the higher their protein concentrations. The relationship between the length and width of trophocytes and the protein concentrations was evident in our experiment. These values differ depending on the location of the fat body. This confirms (cf. Strachecka et al.^34^) that the segmental nature of the apian fat body is not only structural, but primarily pertains to the functions of this tissue and the biochemical and physiological processes that take place in it. Moreover, Strachecka et al.^8,34^ suggest that the compounds that are rapidly metabolized for energy (e.g. glucose and triglycerides) are held in the fat body in the tergites (as opposed to the sternites), especially the third tergite. This tergite is in close proximity to the heart, ostia and body cavities, and the compounds are immediately transferred to the appropriate tissues by the circulatory system. Our study also confirmed that the fat body in the third and fifth tergites is metabolically the most active in the production of glucose, glycogen and triglycerides. However, we demonstrated that not only 1-day-old bees, as described by Strachecka et al.^34^, but also older workers (7- and 14-day-old) were usually characterized by the highest values of these parameters in the third tergite fat body. The highest concentrations of these compounds are noted in queens (compared to workers) because, unlike honey bee workers, this caste has oenocytes in the third tergite. In turn, we have shown that not only the reproductive status but also the diet can influence the biochemical parameters in the fat body from various locations. Very high glucose concentrations were observed in the 14-day-old worker bees fed with sugar candy with the addition of buckwheat and phacelia pollen. The flowering period of these plants is at the end of the summer, when the generation of winter workers appears in a colony. The demand then increases not only for protein (mainly for the preimaginal stages and for “building” the imago fat body) but also for other compounds, including carbohydrates. They are used to obtain energy (e.g. in muscles; to fly and to warm the larvae), for the synthesis of chitin (a major cuticle component) and in other processes^49,50^. Both buckwheat and phacelia provide bees not only with pollen but also with nectar, which is an aqueous solution of sugars, mainly fructose and glucose. Carbohydrates may be derived from the enzymatic digestion of the cytoplasm of pollen grains (both by bee enzymes and enzymes released by microorganisms in the bee’s digestive tract; Fig. 1). Carbohydrates from the digestive tract reach the fat body through the hemolymph, where they are incorporated into metabolic processes^51^. Glucose present in the hemolymph is used primarily in the bees’ metabolic processes. This may explain why its concentration in our study was the lowest in the hemolymph (regardless of the group). In the natural environment, glucose is used up in metabolic processes faster than in the cage environment. Glucose is stored in the form of glycogen in the trophocytes that form the fat body^30,34,52,53^. It is worth noting that hazel pollen, which is one of the first sources of pollen in spring, led to the highest increase in glucose concentration in the hemolymph compared to the control group. Even though the plant is wind-pollinated, it is very often visited by bees as it provides the first pollen available in spring^54^. Hence, spring pollen boosts the bee colony by providing carbohydrate energy, while autumn pollen does so by enabling compounds to be stored in the fat body. Glycogen is referred to as “metabolic security” because it ensures the survival of insects during periods of starvation^55^. The glycogen concentration in the fat body depended on the availability of pollen in the bees’ diet. The highest concentrations were observed in the bees fed with buckwheat, phacelia and goldenrod pollen. These values were always higher in the fat body, especially from the third tergite, compared to the hemolymph. As mentioned above, these types of pollen come from plants that bloom in late summer and in autumn. Therefore, they must play a special role in shaping the fat body in long-lived winter bees whose tasks include the regulation of in-hive temperatures during winter and the initiation of brood rearing in early spring^56^. The concentrations of glycogen and other compounds (e.g. triglycerides) undoubtedly translate into the fat body mass, which is the highest in winter bees. The values of the biochemical parameters of the hemolymph and fat body were the lowest in the bees fed pine pollen in comparison with the other experimental groups. Pollen produced by the pine has a low sugar − 13.92% and protein concentration—13.45%, which it is not preferred by bees^57,58^. Despite the low content of carbohydrates and proteins, this pollen is rich in numerous polyphenolic compounds, e.g. coumaric, methoxycinnamic, and dihydroxydecanoic acids^59^ and its small addition to the diet of insects, especially in monocultures, results in a diversification of nutrients. The phenomenon of dietary diversification can be observed in the pollen mixture from which rapeseed was isolated. In addition to rapeseed, honey bee workers collect pollen from anemophilous plants in order to diversify their diet. After manual sorting, there was still 8% of other pollen grains present, including pine pollen, which we were unable to manually separate due to similar colors or contamination. That is why weeds and other plants growing on floral strips are so important, and bush communities significantly increase the biodiversity in the agricultural landscape^60,61^. The available literature shows that despite large resources of cultivated pollen plants, honey bees willingly visit non-cultivated plants^62^. Scientists report a lack of research that will allow to assess the impact of individual pollen plants on the physiology of insects, including their immunity^13^. Most studies to date have focused on the impact of pollen diets on the development of the hypopharyngeal glands (HPG)^42^. There are relatively few studies on their impact on immunocompetence^12^. Therefore, our research is in line with the latest trends in the field of bee nutriphysiology. Moreover, the variability of protein content in pollen grains depends on the season^63^ and on the number and species of plants present in the area of the apiary. Reducing dietary protein results in higher worker mortality^16^. It has been pointed out that in order to help diversify the bees’ diet with protein, it is important to know the changes in its concentration throughout the season^63^. Our research partially explains this phenomenon, as we have recorded changes in the protein contents and other biochemical parameters in bee tissues depending on the type of pollen. To understand the effect of different pollen types, we selected for research those plants that might be the most important for bees in particular seasons/months and provide a significant proportion of their diet. Although homogenous pollen has a stimulating effect, a pollen combination should produce better results. Frias et al. ^5^ suggest that the extension of life is positively related to the amount of consumed protein contained in pollen, and negatively correlated with the low survival of adult workers whose diet contained pollen from the Asteraceae family. In our research, we also used the pollen of *Solidago* sp., a member of this family. In both biochemical and morphometric studies, goldenrod pollen had a similar impact to summer and autumn pollen (e.g. buckwheat and phacelia). Canadian goldenrod, *Solidago canadensis* L., is classified as an invasive species whose encroachment into new geographical regions has accelerated, thereby decreasing native biodiversity^64^. However, it turns out this plant is highly attractive tohoney bees^65^. Goldenrod flowers at the very end of the beekeeping season, making up 80% of the bees’ diet as compared to other plants^64^. During this period, honey bee workers feed only on goldenrod. In this way, the environment itself provides a mono-diet. In the case of rapeseed, buckwheat, phacelia, soybean and corn, people create large-scale cultures themselves.

Current research shows that bees prefer plants that produce pollen with a higher protein content. Moreover, this preference depends on the ratio of macronutrients contained, not on a single ingredient ^9^. Phacelia is a valuable plant for bees from a physiological perspective. When fed with sugar candy supplemented with phacelia pollen in the cage experiments, our bees had higher concentrations of glucose, glycogen, triglycerides and proteins than the worker bees fed with sugar candy only. A standard, balanced diet for bees in commercial beekeeping continues to be sought to meet the nutritional needs of bees around the world^66^. We propose the use of phacelia as a rapidly-growing plant and a tested “superfood” for pollinating insects.

The nutritional value of pollen lies not only in the proteins but also the fats. Pollen is also the principal dietary source of sterols, triglycerides and phospholipids^67^. Fatty acids are stored in the form of triglycerides as energy reserves for use in the fat body. Reserves are used during winter and in times of famine. This means that fats are gradually released into the hemolymph, which is why their concentration increases with age. It turns out that stress, e.g. in the form of pathogens or an E-field, reduces the level of triglycerides in the hemolymph^68^. Bee colony supplements are administered to increase resistance to pathogens and other environmental factors. Natural supplements counteract the negative effects caused by stressors and, for example, increase the concentration of triglycerides^69^. In our case, the addition of pollen resulted in an increase in the concentrations of triglycerides in all the tissues/tissue locations, especially in the fat body of the third tergite. These results correspond to the results of Strachecka et al.^8^. They showed that the greatest amounts of triglycerides were stored in the third tergite. As compared with spring pollen, summer and autumn pollen caused the greatest increase in triglyceride concentrations, especially in the third tergite. This means that insect-pollinated plants produce pollen that is richer in floral rewards than that of wind-pollinated plants^70^.

Nutrients including proteins, lipids and glycogen are stored in trophocytes. There are differences in the cellular structure of trophocytes and enocytes of the fat body between castes and segments in worker bees^34^. The pollen diet will also be a factor determining the length and width of cells in the fat body. The greatest influence on the growth of trophocytes was exerted by the addition of summer and autumn pollen. In our case, the increase in trophocyte dimensions was associated with a higher concentration of triglycerides and glycogen. The pine turned out to be the most physiologically neutral pollen (neither harmful nor helpful) with respect to trophocyte dimension. Oenocytes in worker bees are present in all segments except tergite 3^34^. All the pollen diets resulted in an increase in the oenocyte diameter. Hence, the pollen diet affects the metabolic activities of oenocyte nuclei. The diameter of oenocytes is not normally distributed. According to the Kruskal–Wallis test, the diameter of oenocytes between the groups is statistically significant (see supplementary material S1).

## Conclusions

We need to know how particular types of plant pollen that dominate the honey bee’s diet influence the physiological parameters of the fat body. This research will enable the development of mixtures from nectar-producing and pollen-producing plants that will meet the nutritional needs of bees.

Further research is needed on the pollen diet of workers in relation to its impact on biochemical parameters, including immunity and ageing-related processes. Healthier bees correspond with higher pollination efficiency and greater yields of honey and other bee products from a colony. This is obviously financially profitable to the beekeeper. Our pioneering research has shown how particular types of pollen from plants blooming from early spring to late autumn influence the morphology of fat body cells in different locations and the concentrations of compounds important for energy metabolism in the hemolymph and fat body of worker bees. Pollen from early spring plants provides bees only with carbohydrates which are quickly used up by their bodies during the rearing of the first generation of summer bees and during the work performed for the colony at that time. However, pollen from plants blooming in late summer and early autumn increases the concentrations of proteins, glycogen and triglycerides in the fat body, especially in the third tergite. Thus, large amounts of compounds are accumulated, which causes the trophocytes to increase in size. It is important to bear in mind that a multi-pollen diet is always more beneficial for the apian body than a mono-diet. However, in order to understand the mechanisms that occur in bees under the influence of pollen, it is important to know the interactions of the particular pollen types.

### Supplementary Information

Supplementary Information.

## Data Availability

Raw data is available from the corresponding author. Anyone can access the raw data after sending a query. Moreover, the raw data is stored in the repository of the University of Life Sciences in Lublin (link URL: https://repozytorium.up.lublin.pl/pl/search_results/5420?q%5Bq%5D=&q%5Bsort_attributes%5D%5Bfield_name%5D=created_at&q%5Bsort_attributes%5D%5Border%5D=desc). Handle https://hdl.handle.net/20.500.12838/5420
