# Supplementary material for: The effect of pollen monodiets on fat body morphology parameters and energy substrate levels in the fat body and hemolymph of Apis mellifera L. workers
Source: Sci Rep. 2024 Jul 2;14:15177. doi: 10.1038/s41598-024-64598-0 (PMC11219722; doi:10.1038/s41598-024-64598-0)
Supplement: Supplementary file 1 — Supplementary Information. [file 41598_2024_64598_MOESM1_ESM.docx]

Suplemmentary material

**The effect of pollen monodiets on fat body morphology parameters and energy substrate levels in the fat body and hemolymph of *Apis mellifera* L. workers**

Maciej S. Bryś^1*^, Bernard Staniec^2^, Aneta Strachecka^1^

**S1. Diameters of oenocyte (μm) in fat body’s from different localization in 1-. 7- and 14-day-old workers (n= 40-55 bees per group) fed sugar candy only (control group) and those fed sugar candy with additional variouadded pollen.**

|  |  |  |  | | **Pollen added to sugar candy** | | | | | | | | | | | |
| --- | --- | --- | --- | --- | --- | --- | --- | --- | --- | --- | --- | --- | --- | --- | --- | --- |
| Day of life  1  7  14 |  | Localization  tissues | **Control** | | **Hazel** | | **Pine** | | **Rape** | | **Buckwheat** | | **Phacelia** | | **Goldenrod** | |
|  |  |  | Mean | SD | Mean | SD | Mean | SD | Mean | SD | Mean | SD | Mean | SD | Mean | SD |
|  | Fat body | Sternite | 30.94 | 7.19 |  |  |  |  |  |  |  |  |  |  |  |  |
|  |  | Tergite 4 | 35.02 | 6.04 |  |  |  |  |  |  |  |  |  |  |  |  |
|  |  | Tergite 5 | 29.65 | 5.36 |  |  |  |  |  |  |  |  |  |  |  |  |
|  |  | Tergite 6 | 28.61 | 7.93 |  |  |  |  |  |  |  |  |  |  |  |  |
|  |  | Tergite 7 | 26.22 | 11.35 |  |  |  |  |  |  |  |  |  |  |  |  |
|  | Fat body | Sternite | 36.06 | 5.74 | 38.92 | 4.45 | 28.77 | 3.96 | 29.06 | 3.15 | 39.36 | 2.57 | 27.09 | 7.01 | 33.70 | 2.43 |
|  |  | Tergite 4 | 35.26 | 6.42 | 25.13 | 4.68 | 34.68 | 3.15 | 35.31 | 2.93 | 35.36 | 7.42 | 30.74 | 3.83 | 26.70 | 4.32 |
|  |  | Tergite 5 | 31.15 | 4.78 | 31.18 | 1.75 | 29.18 | 2.02 | 27.59 | 1.87 | 29.75 | 1.84 | 34.51 | 4.17 | 24.70 | 8.22 |
|  |  | Tergite 6 | 30.15 | 8.33 | 34.98 | 10.47 | 29.45 | 1.17 | 29.45 | 1.52 | 33.29 | 5.19 | 36.80 | 5.30 | 23.22 | 7.34 |
|  |  | Tergite 7 | 27.72 | 9.99 | 27.97 | 2.76 | 31.62 | 5.41 | 31.62 | 5.50 | 42.79 | 2.47 | 37.14 | 5.35 | 31.26 | 3.32 |
|  | Fat body | Sternite | 35.10 | 7.72 | 50.77 | 8.49 | 34.23 | 3.86 | 30.24 | 1.49 | 43.64 | 4.84 | 104.8 | 12.90 | 38.48 | 8.25 |
|  |  | Tergite 4 | 36.42 | 6.39 | 33.34 | 3.86 | 45.77 | 11.03 | 29.14 | 1.89 | 38.37 | 6.15 | 97.35 | 16.78 | 78.87 | 13.24 |
|  |  | Tergite 5 | 31.70 | 4.90 | 51.47 | 5.06 | 31.28 | 6.57 | 30.50 | 1.68 | 34.97 | 4.45 | 71.48 | 10.42 | 34.88 | 5.81 |
|  |  | Tergite 6 | 30.98 | 8.01 | 48.77 | 3.54 | 33.46 | 5.49 | 32.51 | 5.54 | 41.75 | 5.37 | 51.54 | 9.36 | 27.57 | 1.27 |
|  |  | Tergite 7 | 31.52 | 8.19 | 55.40 | 8.34 | 31.15 | 5.017 | 27.96 | 2.48 | 45.09 | 7.19 | 52.04 | 8.18 | 59.06 | 11.42 |

Kruskal–Wallis test, the diameter of oenocytes between groups is statistically significant
H = 899.817, df= 6, p<0.005; oenocyte diameter and age: H = 522.615, df = 2, p < 0.05; oenocyte diameter and location H = 238.083, df= 4, p<0.05.
